# An automated daily QA strategy for dosimetry and imaging guidance check of a novel PET/CT ring gantry linac

**DOI:** 10.1002/acm2.70309

**Published:** 2025-11-18

**Authors:** Sagar Ghimire, Yang Kyun Park, Grant Gibbard, Jun Tan, Thomas I. Banks, Chenyang Shen, Rameshwar Prasad, Tingliang Zhuang, Andrew Godley, Steve B. Jiang, Bin Cai

**Affiliations:** ^1^ Department of Radiation Oncology University of Texas Southwestern Medical Center Dallas Texas USA; ^2^ RefleXion Medical Hayward California USA; ^3^ Department of Radiation Oncology Mayo Clinic Jasonville Florida USA

**Keywords:** Daily QA, image‐guided radiotherapy, PET‐linac, RefleXion X1

## Abstract

**Background:**

The RefleXion X1 is a novel PET/CT‐guided ring gantry linac that introduces unique geometry and workflow compared to conventional systems. Existing QA guidelines (AAPM TG‐142, 148, 306) cover standard linacs and tomotherapy but do not address this platform's needs. Therefore, an automated daily QA solution is required to ensure accurate output, imaging guidance, and couch positioning, which motivated the development and evaluation of the workflow presented in this study.

**Purpose:**

To develop and evaluate an automated, efficient, and comprehensive daily quality assurance (QA) workflow for a novel PET/CT‐guided ring gantry linear accelerator (RefleXion X1), incorporating tests for dosimetry, imaging guidance, and couch positioning accuracy.

**Methods:**

An IMRT QA plan was designed following the CT scan of the TomoDose 2D diode detector array (Sun Nuclear, Melbourne, FL) within the RefleXion treatment planning system (TPS). Several spherical targets of varying sizes were contoured to conform the dose distribution to these targets and evaluate beam output, laser localization, profile constancy, and CT‐MV isocenter coincidence. Linear models correlating spatial location of dose peak with intentional shifts in different directions were developed and integrated into an in‐house analysis software, iQA, written in C#. The software was validated for robustness and successfully implemented in clinical practice.

**Results:**

Validation with 42 intentional shifts showed submillimeter accuracy, with mean deviations of 0.2 mm (X), 0.1 mm (Y), and 0.79 mm (Z), and *R* ^2^ values ≥ 0.98. Over 1 year, daily QA data showed stable output constancy (1.006 ± 0.016), left/right target dose constancy (1.007 ± 0.005 and 0.996 ± 0.004), and consistent CT‐MV isocenter offsets (–0.335 ± 0.23 mm in X, 0.19 ± 0.19 mm in Y, and –0.544 ± 0.36 mm in Z). The software also detected a gradual output drift in between, prompting a timely linac adjustment.

**Conclusion:**

This fully automated, single‐delivery QA workflow enables efficient and robust evaluation of multiple IGRT performance parameters. It improves standardization, reduces human error, and reliably monitors long‐term machine stability. The tools and methodology may be adopted by other clinics seeking a practical and automated daily QA solution. Future developments will focus on incorporating PET QA and rotational couch validations to further enhance the system's comprehensiveness and adaptability.

## 1 INTRODUCTION

Biologically guided radiotherapy (BgRT) has gained significant attention from the radiation oncology community due to its potential to incorporate biological information into radiotherapy.^1^ More recently, a commercial platform, RefleXion X1 (Hayward, CA), was released to achieve this goal by integrating a Positron Emission Tomography (PET) scanner with a compact ring gantry linear accelerator.^2^, ^3^, ^4^ Equipped with an on‐board kV fan beam CT (kVCT) system,^5^ a six‐degree‐of‐freedom (6DOF) couch and a binary multi‐leaf collimator (MLC), the X1 machine is capable of delivering 6FFF beam treatment plans in an axial delivery fashion.^6^ The kVCT provides high‐quality CT images for Image Guided Radiation Therapy (IGRT) localization and the 6DOF couch provides more flexibility for patient alignment. The PET imaging module is designed to guide BgRT treatments. Due to the fast imaging and tracking requirement from the PET guidance, the entire system rotates at a rapid speed of 60 rotations per minute (RPM), with the MLC opening or closing within 10 ms. Since this is a novel treatment modality, challenges exist with operation of the ring gantry linac and the current workflow. For example, first, there is no light field or cross hair on this modality to easily spot the post couch shift isocenter check; second, since it is an axial delivery, the first couch position after kV imaging alignment is not necessarily the treatment isocenter; lastly, the couch cannot easily be returned to the setup position for re‐verification after the completion of the treatment.

National guidelines such as those developed by the American Association of Physicists in Medicine (AAPM) Task Groups (TG) 142, 148, and 306 provide general guidance and recommendations on routine QA tests for L‐shaped linear accelerators or helical tomotherapy machines.^7^, ^8^, ^9^ Daily output, imaging guidance and couch positioning/re‐positioning tests are major components of any daily QA program for an IGRT machine. Typically, the daily QA is performed by radiation therapists and ideally, should be performed in treatment mode with minimal human interactions and automated analysis to reduce errors and improve efficiency. As the treatment system becomes more complex, a robust and efficient daily QA approach is critical to detect any sub‐optimal machine performance in a timely manner. Since the release of X1 machine, only a few articles were published to describe and characterize the machine performance.^5^, ^6^, ^10^, ^11^ However, none of these articles focuses on establishment of an automated daily QA program. In this study, based on the recommendations of AAPM TG‐142, 148, and 306, we developed an end‐to‐end daily QA strategy to test the performance of the Food and Drug Administration (FDA)‐approved IGRT components of the machine, utilizing a 2D array detector and an in‐house automated analysis software.

The aim is to provide “one‐button‐ click” solution after detector set‐up to efficiently evaluate three aspects of machine performance:(1) Daily output consistency; (2) kVCT imaging guidance functionality; and (3) couch positioning/repositioning accuracy. This work only focusses on daily QA on kVCT guided IGRT workflow e.g., IMRT, SBRT. The daily QA of PET imaging component is not includes in this study which will be discussed in future publications.

## 2 MATERIALS AND METHODS

### 2.1 RefleXion system, IGRT workflow and DailyQA 2D detector

**FIGURE 1:**
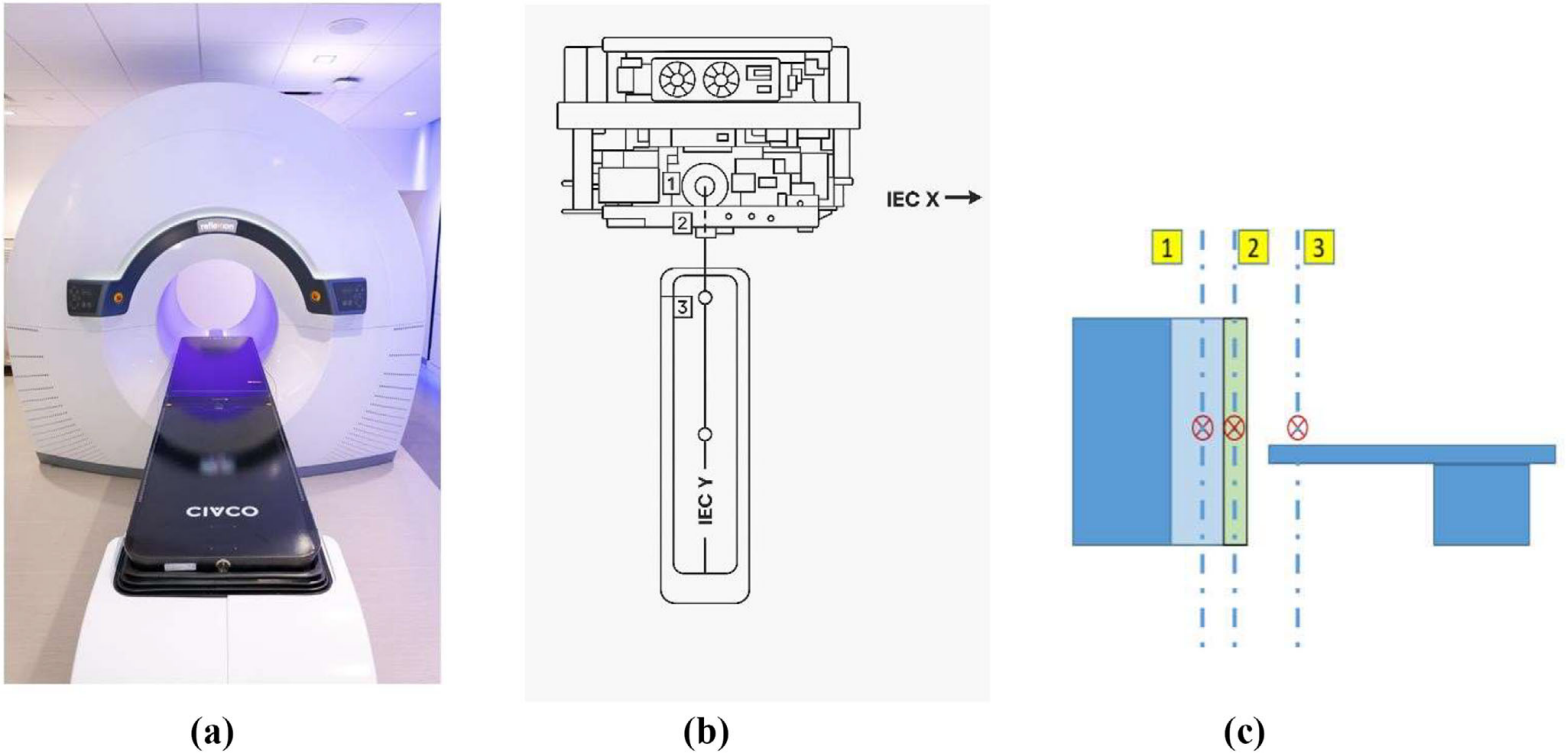
(a) Photograph of the RefleXion X1 machine installed in the treatment room, showing the ring gantry design with an integrated patient couch. (b) Top‐view schematic of the X1 machine illustrating the relative positions of the three distinct isocenters used in clinical operation.^11^ (c) Cross section view of the system with three isocenters. 1—treatment isocenter; 2—imaging isocenter; and 3—setup isocenter.

An overview of RefleXion X1 system^2^ is shown in Figure 1. Figure 1a shows the actual picture of the machine installed in the facility vault, Figure 1b shows the schematic diagram of the machine components, and Figure 1c shows the schematic diagram of the machine isocenters. The MV linac and PET detectors sits on the same ring and the kVCT system sits on a separate ring. In Figure 1c, Label 1 indicates the treatment isocenter, Label 2 represents the kVCT imaging isocenter and Label 3 indicates the external setup isocenter by external laser. The IGRT workflow starts with phantom setup at external setup‐isocenter position indicated by external laser. Once setup position is confirmed, the system sends the phantom to the couch position of inferior end of kVCT imaging extent. A helical CT scan is then performed based on selected imaging protocol. Once kVCT is completed, the system reconstructs high quality CT images and 3D registration is performed at the treatment console. Couch position is adjusted based on the image registration results and the system sends the patient or phantom to the first couch position of treatment.

In this study, we used a two‐dimensional (2D) dose measuring device, TomoDose (Sun Nuclear, Melbourne, FL) array for dosimetric measurement. The TomoDose device contains a total of 223 diode detectors distributed along the X and Y directions and provides sufficient resolution to measure radiation from field sizes as large as 40 cm × 3 cm, as available on the RefleXion system.

### 2.2 Daily QA treatment plan design

**FIGURE 2:**
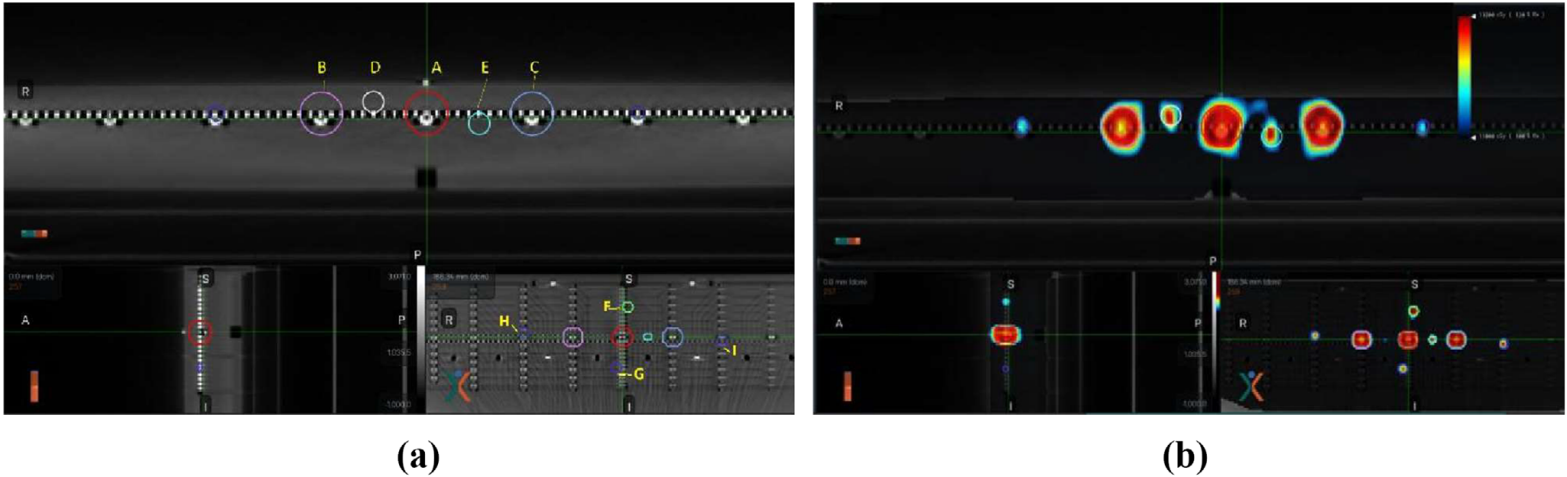
(a) Contours (A–I) to conform the dose map at distinct location of detectors. (b) Calculated dose distribution map on the phantom.

The TomoDose detector array was scanned with simulation CT using 2 mm slice thickness. Multiple contours were drawn and used as dose painting and evaluation structures as shown in Figure 2a. The circle A is used for output evaluation; circles B and C are used for profile constancy check; Circle D, E, F, G, H, and I are used for offset evaluation to check imaging‐treatment geometry coincidence in conjunction with couch shift. An IMRT QA plan was generated with appropriate constraints to form a unique dosimetric distribution^12^ in the RefleXion treatment planning system (TPS). The final dose distribution is shown in Figure 2b. By comparing the shape, intensity, and location of beam profiles with baseline, dosimetric parameters for MV beam as well as verification of image guidance and couch positioning accuracy can be derived. To build and validate a shift‐detection model, a series of intentional positional shifts were incorporated into the delivery of this plan. These shifts were used to correlate dose peak ratios with known offsets; further details are described in Section 2.6.

### 2.3 Daily QA workflow

**FIGURE 3:**
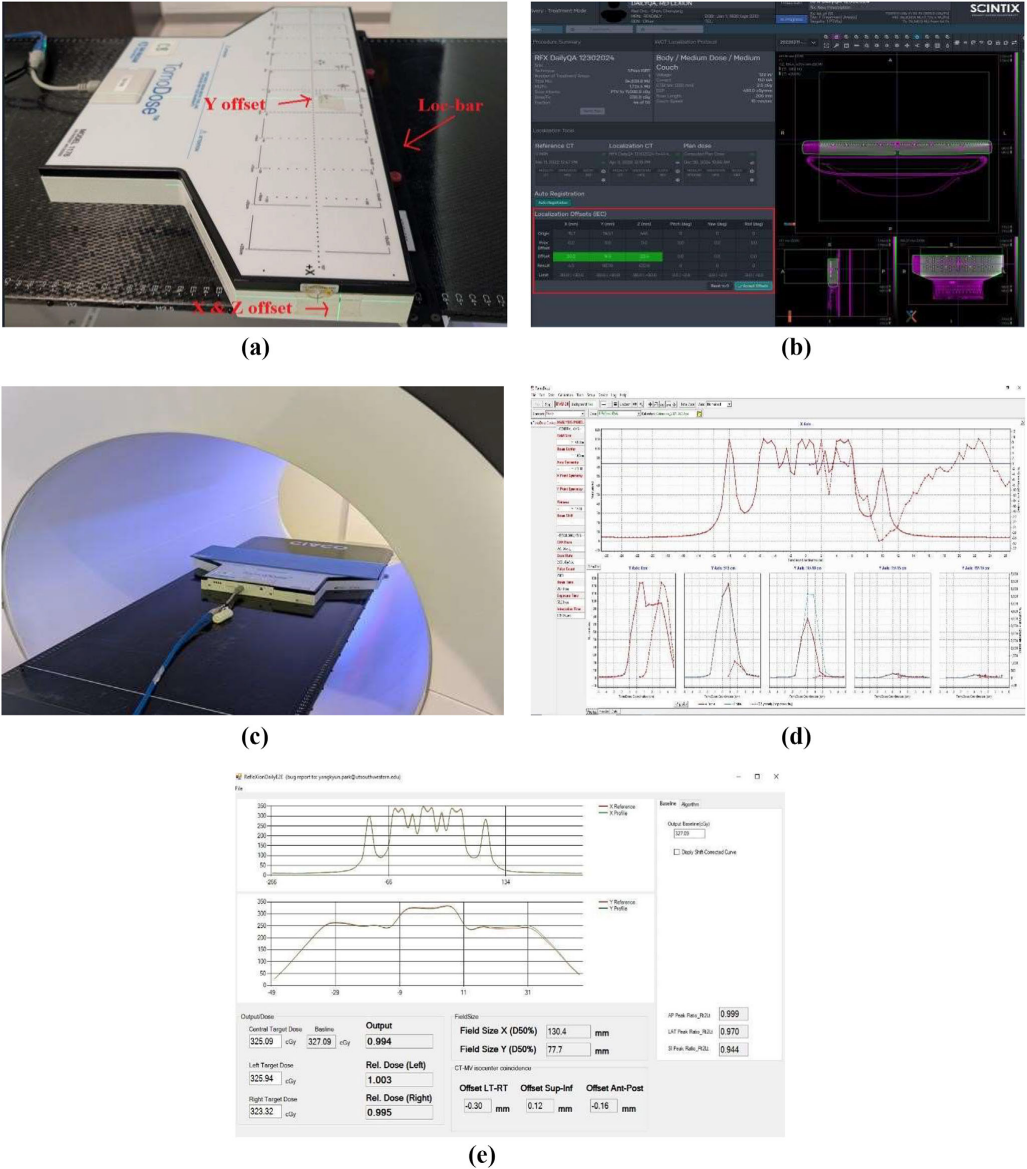
(a) Step 1: Setting up Tomodose phantom to laser pre‐set offset. (b) Step 2: kV‐CT alignment and recording the shifts – shifts are indicated inside red box. (c) Step 3: Applying the couch shift and moving to treatment position for plan delivery (d) Step 4: Concurrent Tomodose measurement during the plan delivery. (e) Step 5: Recording and analyzing Tomodose measurement output file in iQA software.

The overall workflow for the DailyQA procedure is described in five easy and concise steps as shown in Figure 3. TomoDose phantom is first set up on the couch using lasers aligned to pre‐determined markers that correspond to known offset values along the Left–Right (LT–RT) axis (X), the Superior–Inferior (SUP–INF) axis (Y), and the Anterior–Posterior (ANT–POST) axis (Z), as shown in Step 1 of Figure 3a. To further improve the setup consistency, the phantom is anchored adjacent to the index bar at a predefined location corresponding to couch index position “C8”.

A 0.5 cm solid water slab was added as build up followed by confirming the couch position and the acquisition of localization kVCT image. The acquired image is registered to the reference CT to determine the necessary couch positions as shown in Step‐2 in Figure 3b. This 3D shift is compared to the pre‐determined offset to assess the setup accuracy of the lasers in LT‐RT, SUP‐INF and ANT‐POST directions, and the offsets are applied to reposition the phantom. After repositioning, phantom is moved to the treatment position as shown in Step‐3 in Figure 3c. The system then delivers the IMRT QA plan with the resulting 2D dose distribution captured using the TomoDose software as represented in Step‐4 in Figure 3d. Finally, the measurement output file from the TomoDose software is loaded into the in‐house software, IntelligentQA (iQA), for analysis.

### 2.4 Daily QA analysis software design

To make the entire process fully automated and to assist with TomoDose file analysis, iQA software was written in C#. This software utilizes the shape, intensity, and location of landmark features of the baseline beam profile of the daily QA plan to that of the TomoDose measured profile, and thus calculates the dosimetrical and geometrical parameters such as beam center output constancy, off‐axis output constancy, field size, and CT‐MV isocenter offsets.

**FIGURE 4:**
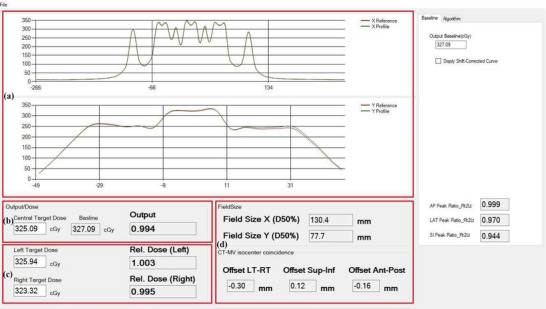
Graphical user interface of the in‐house software iQA represented in four primary modules (a–d).

Figure 4 shows the graphical user interface of iQA software which has four primary modules. **Module‐A** represents the beam profile comparisons where top figure of the module represents the X reference profile compared to the currently measured profile and the bottom figure represents the similar comparison for the Y profile. **Module‐B** represents the dose values measured at the center peak of the currently measured profile in cGy unit, with baseline value displayed adjacent to it. This is used to calculate and display the machine daily output factor. **Module‐C** shows the left and right target dose to calculate the lateral profile constancy in cGy with output factors adjacent to the respective measurements. **Module‐D** provides the data regarding the field size constancy and CT‐MV isocenter offset values, both in millimeters.

### 2.5 Data processing

iQA software utilizes the method of identifying the intensity and spatial location of dose peaks in the X and Y profiles, as seen in Figure 4, measured using a 2D detector array, to quantify output constancy, profile consistency, and phantom offset.

**FIGURE 5:**
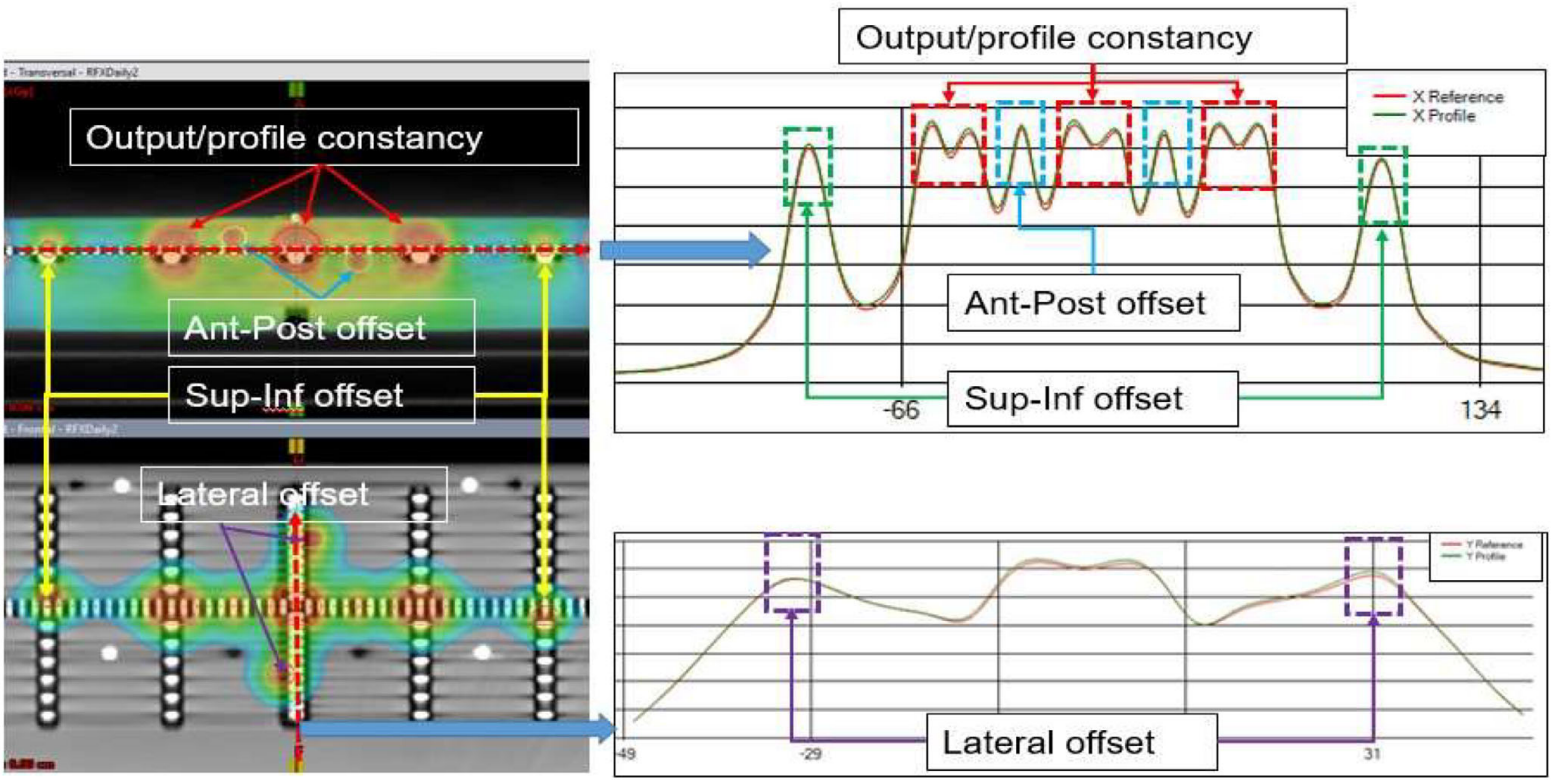
Profile based calculation of output, profile constancy, and CT‐MV isocenter offsets.

Figure 5 illustrates the overall process for calculating these QA metrics. The image in the left displays the computed 2D dose distribution map within the TomoDose phantom in the TPS, while the right panel presents the corresponding profile analysis. In this figure, the red dotted box represents dose peaks used to calculate output constancy and off axis dose constancy, the blue dotted box indicate the dose peaks used to calculate the ANT–POST offset, the green box highlights dose peaks used to calculate the SUP–INF, and the purple box marks the LT–RT offset. For the field size constancy check, the full width at half maximum (FWHM) of a specific region of interest was used.

### 2.6 Dose ratio and shift correlation model

**FIGURE 6:**
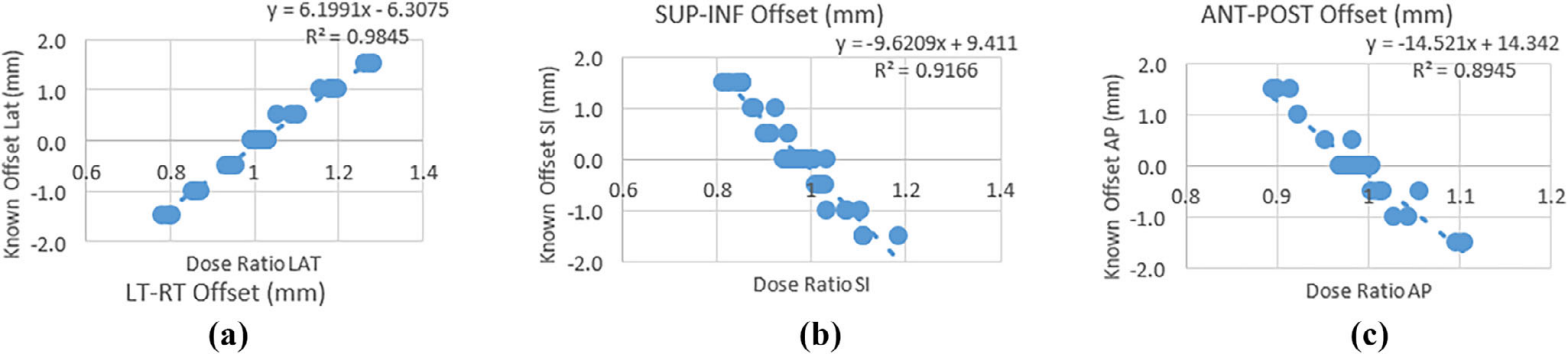
(a) Established linear fit model showing the relationship between dose ratio and positional offset in the left–right (LT–RT) direction. The slope, intercept, and coefficient of determination (R^2^) indicate strong linear correlation. (b) Similar linear relationship between dose ratio and positional offset in the superior–inferior (SUP–INF) direction, with the negative slope indicating inverse correlation. (c) Linear fit between dose ratio and positional offset in the anterior–posterior (ANT–POST) direction, showing a moderate to strong correlation.

To develop and validate the models for estimating positional shifts using the TomoDose phantom, the designed IMRT QA plan was delivered with 43 combinations of intentional shifts applied individually and in combination, in the LT–RT, SUP–INF and ANT–POST directions. The range of shifts varied from –1.5 mm to +1.5 mm in 0.5 mm increments. From these deliveries, a correlation model between the ratio of dose peaks and the shifts were established for each directional axis. As shown in Figure 6a–c, three separate linear fits were established with *R* ^2^ values exceeding 0.89, enabling accurate estimation of shifts from dose ratios.

These models were then integrated into the in‐house iQA software. The validation of the software was carried out to evaluate the robustness and reliability by comparing the measured offsets to the ground truth values.

Additionally, this QA process was repeated regularly over the course of a year, with results analyzed to assess long term consistency and accuracy.

## 3 RESULTS

The integration of the iQA software into daily the QA procedure has streamlined the process, making it more efficient, and easy to monitor the beam output, profile constancy, and CT‐MV isocenter offsets. The entire process takes less than 30 min to complete. The software was released only after thorough testing and validation of the model implemented in iQA. The results are presented below:

### 3.1 Daily QA results

**FIGURE 7:**
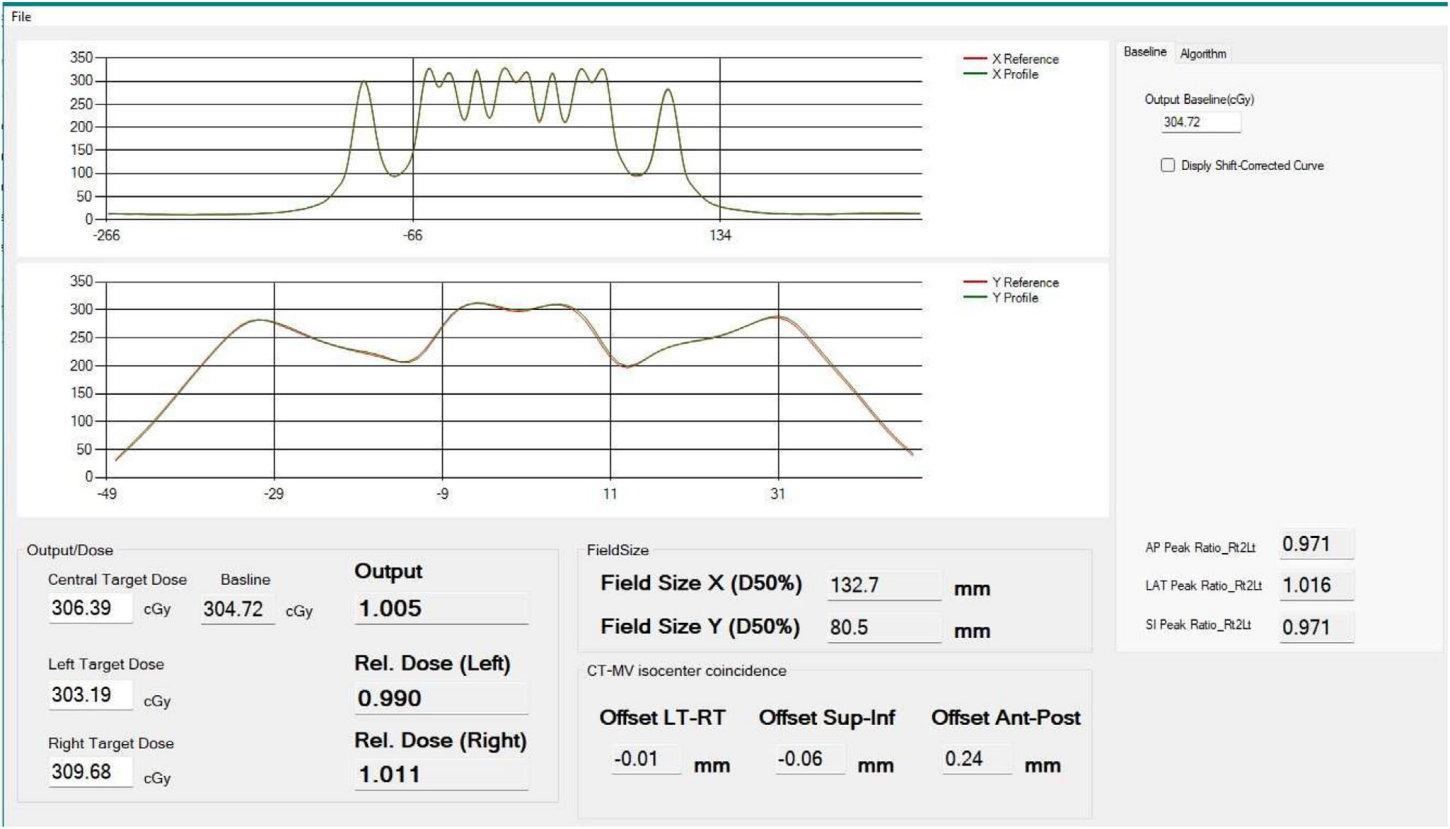
Baseline profile comparison to the day to day profile using the Tomodose measurement file.

Figure 7 provides an overview of the data analysis and results obtained from the daily profile using the iQA software. The software automatically compares the daily beam profile to a predefined reference profile.

The output is calculated using the central dose measured on the day—for example, 306.39 cGy—compared to the baseline dose of 304.72 cGy, yielding the daily output of 1.005. Similarly, the X and Y profile constancy values as calculated by iQA are 132.7 mm and 80.5 mm, respectively. The CT‐MV isocenter offsets were determined with submillimeter precision: −0.01 mm (LT–RT), ‐0.06 mm (SUP–INF), and 0.24 mm (ANT–POST), as shown in Figure 7.

### 3.2 Validation tests

**FIGURE 8:**
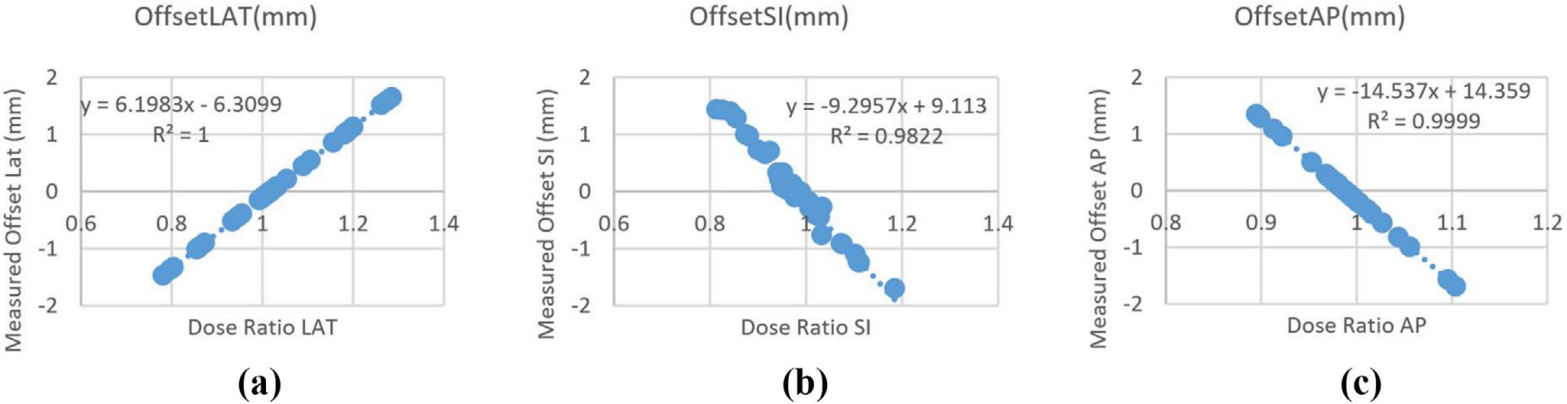
(a) LT–RT shifts measured by IQA. (b) SUP–INF shifts measured by IQA. (c) ANT–POST shifts measured by IQA.

The validation tests for the software were performed using known shifts values ranging from −1.5 mm to 1.5 mm, as shown in Figure 8. Figure 8a shows the iQA‐calculated CT‐MV offset in the LT‐RT for known shifts applied in 0.5 mm increments plotted against dose peak ratio. The resulting linear fit demonstrated excellent agreement, with an *R* ^2^ value of 1.000, indicating high accuracy in detecting X‐directional shifts. Similarly, for SUP‐INF directional shifts, the linear model showed very good agreement with an *R* ^2^ value of 0.9822, as shown in Figure 8b. The ANT‐POST direction shifts yielded comparable results, as illustrated in Figure 8c with *R* ^2^ of 0.9999.

**FIGURE 9:**
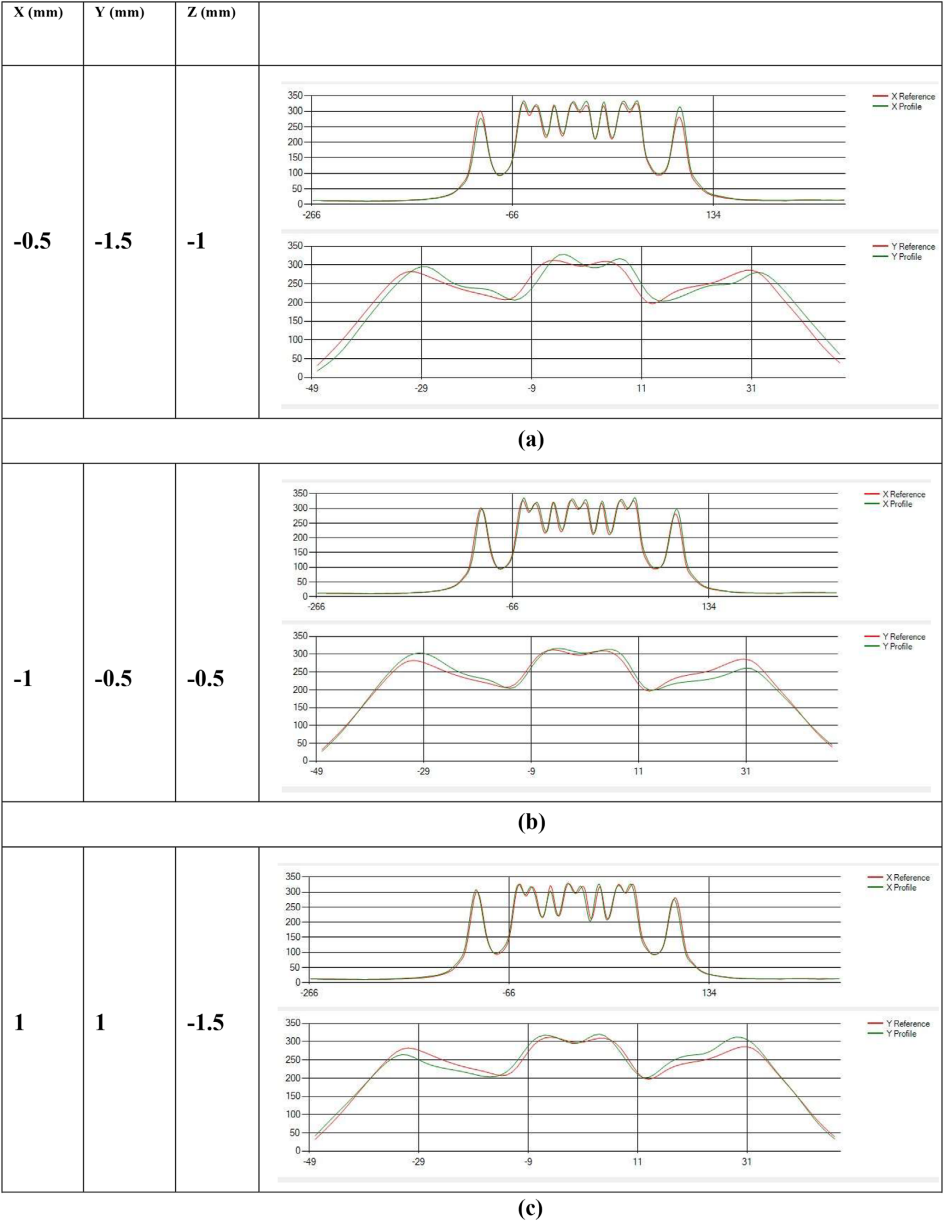
(a) Baseline profile compared to the shifted profile in *X* = −0.5 mm, *Y* = −1.5 mm, *Z* = −1 mm. (b) Baseline profile compared to the shifted profile in *X* = −1 mm, *Y* = −0.5 mm, *Z *= −0.5 mm. (c) Baseline profile compared to the shifted profile in *X* = 1 mm, *Y* = 1 mm, *Z* = −1.5 mm.

The validation tests results can also be visualized thorough the profile comparisons in the iQA software. In Figure 9a, known shifts of *X* = −0.5, *Y* = −1.5, and *Z* = −1 mm were applied to the TomoDose phantom during the positioning step. The iQA displays the offset profile (Green), which is visually noticeable from the baseline profile (Red), in X and Y directions. Figure 9b and Figure 9c show similar representative offsets values applied in X, Y and Z combinations. The validation tests were repeated for more than 42 times across all the directions within a reasonable range of shifts. The iQA software was able to accurately calculate these shifts as summarized in Table 1. The mean deviation from the expected shift was 0.2 mm for the LT–RT direction, 0.1 mm for the SUP–INF direction, and 0.79 mm for the ANT–POST direction. The corresponding standard deviations were 0.18 mm, 0.09 mm, and 1.09 mm, respectively. Table 2 summarizes all the components of the daily QA that were monitored and recorded in this study.

**TABLE 1:** Table summarizing the mean deviation and standard deviation values calculated from the offsets calculated by iQA software, of all 42 validation tests across all the direction of left–right, superior–inferior and anterior–Posterior shifts.

	Left–Right offsets (Δ mm)	Superior–Inferior offsets (Δ mm)	Anterior–Posterior offsets (Δ mm)
Mean	0.20	0.10	0.79
SD	0.18	0.09	1.09

**TABLE 2:** Table summarizing all the QA parameters examined in this study along with their baseline values and mean and the standard deviations during the study period.

QA parameters		Baseline values	Mean	STD
Laser Localization	Left–Right	20 mm	20.18 mm	0.26 mm
	Superior–Inferior	15 mm	14.61 mm	0.31 mm
	Anterior–Posterior	−22 mm	−22.31 mm	0.45 mm
Output Constancy		1	1.006	0.016
Offset Target Dose	Left Target	1	1.007	0.005
	Right Target	1	0.996	0.004
Field Size	X	130 mm	130.36 mm	0.12 mm
	Y	77.5 mm	77.38 mm	0.15 mm
CT‐ MV Offsets	Left–Right	0	−0.335 mm	0.23 mm
	Superior–Inferior	0	0.19 mm	0.19 mm
	Anterior–Posterior	0	−0.544 mm	0.36 mm

### 3.3 Daily laser localization accuracy trend

**FIGURE 10:**
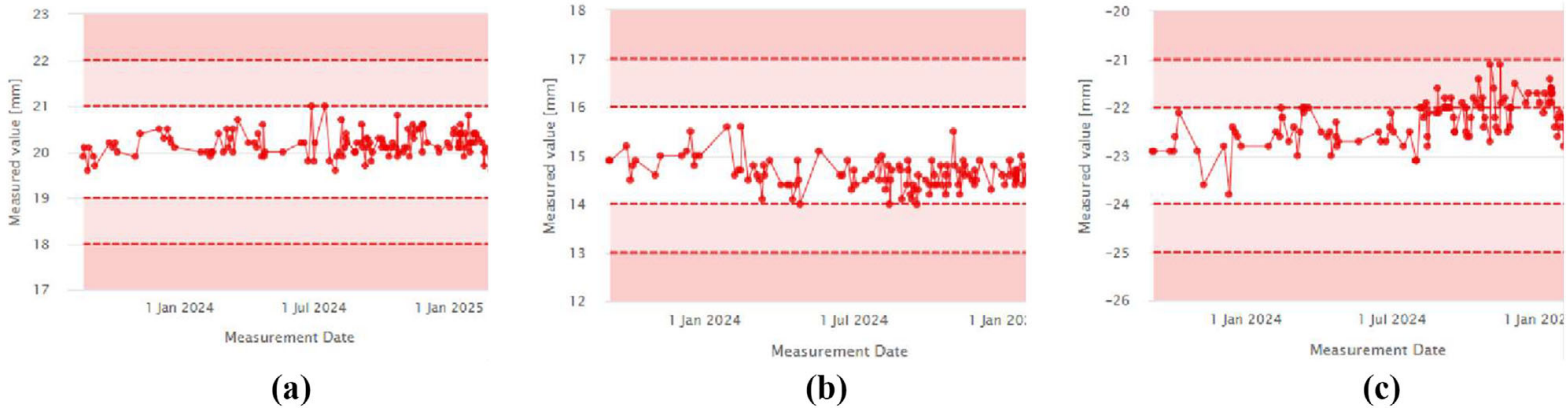
(a) Laser localization offset‐x, (b) Laser localization offset‐y, (c) Laser localization offset‐z.

Figure 10 shows the trend of laser localization values measured over a period of more than one year. The daily values, compared to baselines of 20 mm for lateral (LT–RT), 15 mm for superior‐inferior (SUP–INF), and –22 mm for anterior‐posterior (ANT–POST) lasers, are shown in Figure 10a–c, respectively. The measured averages during this period were 20.18 ± 0.26 mm (LT–RT), 14.61 ± 0.31 mm (SUP–INF), and –22.31 ± 0.45 mm (ANT–POST). While the ANT–POST laser showed a slight deviation from the baseline, it remained within the warning threshold. A laser adjustment was performed during monthly QA in response to the observed drift.

### 3.4 Output constancy trend

**FIGURE 11:**
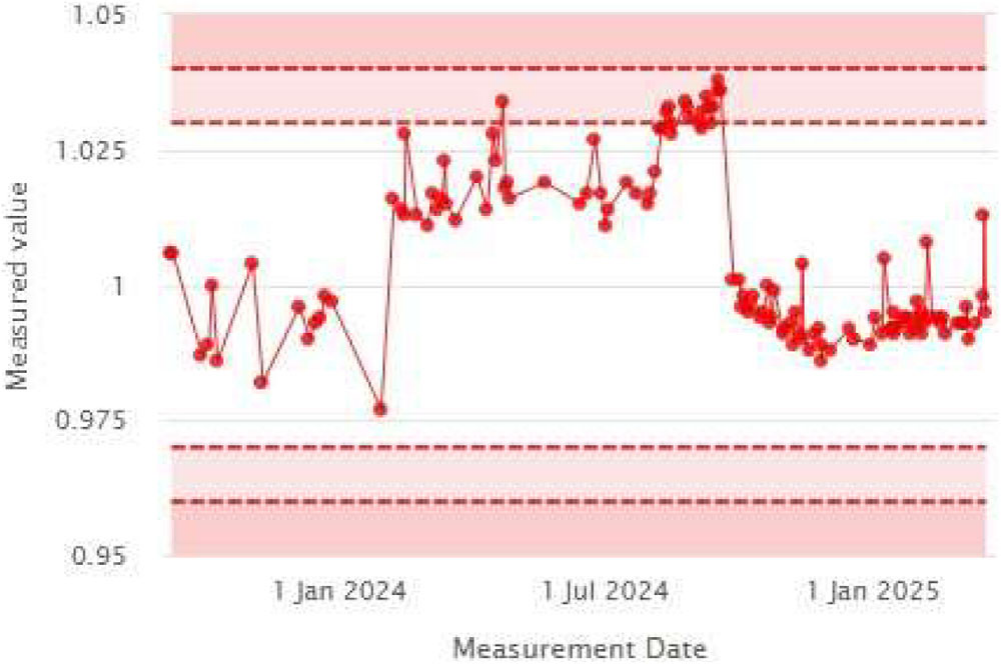
Output trend.

The daily output trend, monitored using data from the iQA software, is shown in Figure 11, with an overall measurement accuracy of 1.006 ± 0.016. Notably, iQA detected a gradual increase in machine output between February and August 2024. This trend led to a linac output adjustment, highlighting the accuracy and clinical value of iQA as an integral component of the daily QA workflow.

### 3.5 Offset target dose constancy and field size trend

**FIGURE 12:**
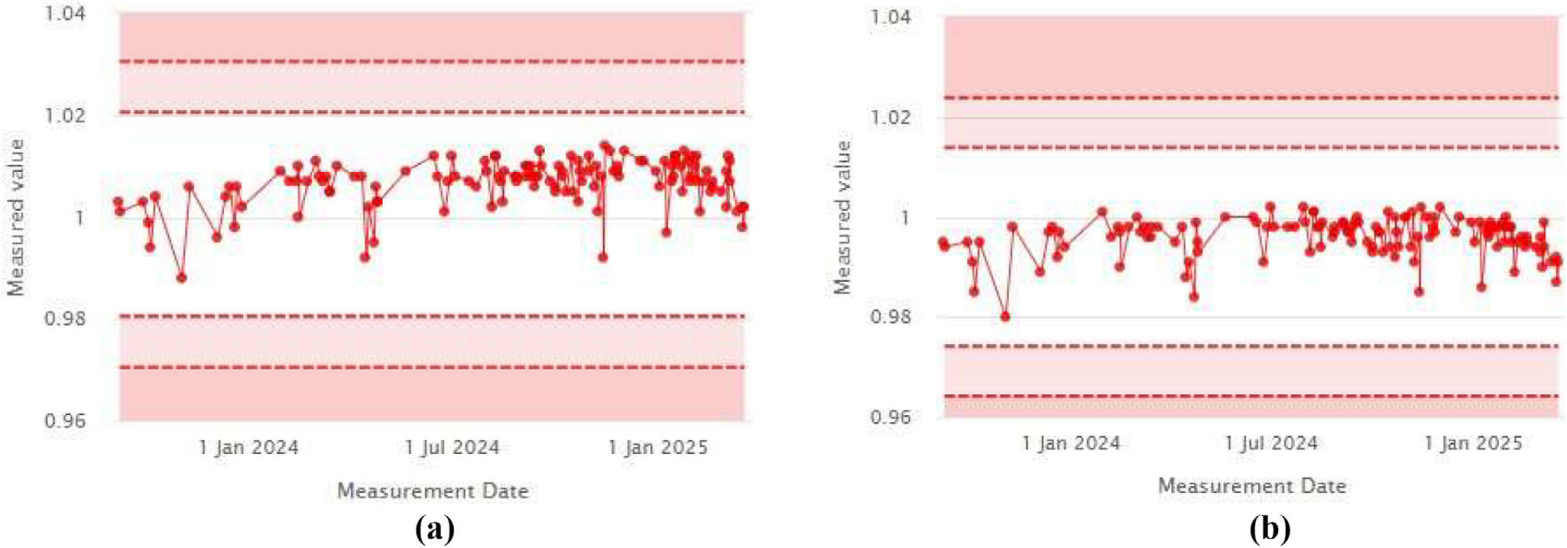
(a) Relative dose (left). (b) Relative dose (Right).

In addition to the output trend, the target offset dose and X–Y field size were monitored over the course of one year. Figure 12a, b show stable trends in the target offset dose measured at the left and right peaks, as calculated by the iQA software. The constancy of the offset dose was maintained at 1.007 ± 0.005 (left) and 0.996 ± 0.004 (right), demonstrating strong dose reproducibility.

**FIGURE 13:**
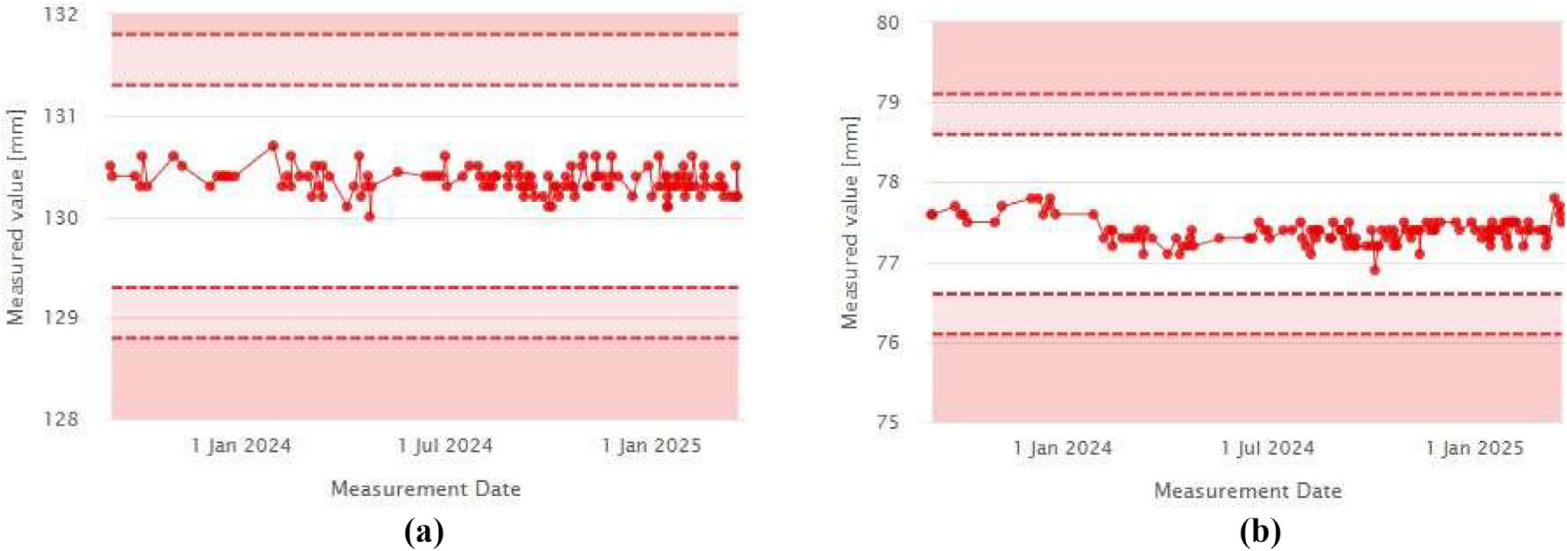
(a) Field size X. (b) Field size Y.

Field size measurements in both X and Y directions also remained consistent throughout the monitoring period, as shown in Figure 13a, b. The measured field sizes were 130.36 ± 0.12 mm (X) and 77.38 ± 0.15 mm (Y), closely matching the respective baseline values of 130 mm and 77.5 mm.

### 3.6 CT‐MV isocenter coincidence trend

**FIGURE 14:**
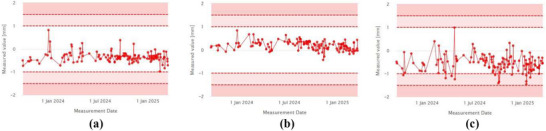
(a) LT–RT offset, (b) SUP–INF offset, (c) ANT–POST offset.

Another key component of the daily QA workflow is the CT‐MV isocenter offset, which was recorded and tracked throughout the year. As shown in Figure 14a, b, the isocenter offsets remained stable and consistent, with average values of –0.335 ± 0.23 mm for the X direction and 0.19 ± 0.19 mm for the Y direction.

The anterior–posterior (ANT–POST) isocenter offset, shown in Figure 14c, remained slightly below zero with an average of –0.544 ± 0.36 mm. These results highlight both the sensitivity of the iQA software in detecting small setup deviations and its reliability in consistently tracking system performance over time.

## 4 DISCUSSION

In this work, a novel approach to perform daily QA for IGRT tests on PET linac has been proposed. The design of this daily QA workflow has been established to incorporate routine DailyQA tests typically performed on L‐shaped or helical tomotherapy machines, as recommended in AAPM TG‐142, TG‐148 and TG‐306. This “one‐button‐click” QA solution was implemented to capture all essential daily QA metrics—laser alignment accuracy, couch positioning and repositioning, CT‐MV imaging coincidence, and beam output consistency—within a single plan delivery using a custom IMRT QA plan. The established workflow has been used daily and can be completed in under 30 min, compared to significantly longer durations required by conventional methods. This was achieved using 2D TomoDose detector, which provides adequate resolution, stability, and field coverage for the RefleXion system's treatment geometry.

The workflow was developed by designing a daily QA plan with multiple targets to evaluate both geometric and dosimetric accuracy. A linear model was established to correlate dose peak ratios with known shifts and integrated into the iQA software for automated offset estimation. The system's robustness was confirmed through over one year of clinical data monitoring.

A major advantage of this approach is its efficiency. One plan is capable of evaluating multiple fronts—laser localization, couch positioning and repositioning accuracy, linac output stability, and functionality of IGRT imaging guidance. This consolidated design addresses many of the practical constraints observed in daily clinical workflows and reduces the cumulative time spent on QA procedures. Compared to QA guidelines such as TG‐142 and TG‐148, which involve independent checks for imaging, dosimetry, and mechanical subsystems, our method achieves all these evaluations in a single delivery with minimal manual intervention. While maintaining the same staffing level and test accuracy, the automated workflow completes the tests in under 30 min, compared to at least 45 min for the equivalent manual procedures, representing a substantial time savings. Another advantage being standardization and reduction of human error. Once the phantom is set up, all remaining steps—from image acquisition to delivery and analysis—are automated. This limits variability due to user interpretation and experience. Previous works on similar daily QA workflow has been presented using flat panel detector on conventional linacs in combination with EPID and on board imaging.^13^, ^14^ These studies demonstrated the benefits of removing the subjectivity associated with user error during routine QA, thereby enhancing reproducibility and confidence in the results. Our workflow follows this same philosophy, offering a robust and repeatable process for evaluating system integrity. Finally, the workflow enables automation, integrating software tools that directly analyze detector data, apply pre‐established models, and giving us the desired outputs. This not only improves throughput but also enables real‐time flagging of deviations. As demonstrated in this work, over the course of year, the system was sensitive enough to detect gradual drifts in output that led to linac output adjustment, reinforcing its value as an early‐warning tool. Despite its strengths, the current implementation has some limitations. First, PET imaging QA, currently performed as a separate procedure, has not yet been integrated into the automated daily QA workflow. As the PET guidance system is central to the RefleXion platform, ensuring its image quality and accurate PET–MV registration is essential for maintaining clinical performance. To address this, we are developing an extension to the current framework that will incorporate a cubic phantom with an embedded point source, enabling objective evaluation of PET image quality metrics and PET–MV co‐registration accuracy. This integration will allow for streamlined, reproducible PET QA within the same automated process, thereby expanding the comprehensiveness of daily system verification. Second, while the system is largely automated, it still requires manual setup of the TomoDose phantom. The accuracy of the QA outcome still depends on consistent phantom alignment and stability of the detector array. So far, our long term data suggests strong stability, but the manual setup still remains a potential source of error. Third, although the RefleXion system supports a 6D couch, the current automated workflow validates only 3D translational shifts. To expand this capability, intentional known rotations in pitch, roll, and yaw can be introduced during the initial setup, with subsequent verification of couch alignment using imaging guidance. This approach would enable direct assessment of rotational correction accuracy, thereby providing a more comprehensive QA of the IGRT system. Lastly, this workflow represents an end‐to‐end test—meaning it evaluates the entire imaging‐to‐treatment chain as a whole. While this is effective for routine performance tracking, it can limit the ability to localize specific failure modes when deviations occur.

In future versions, modular diagnostic tools could be developed to decouple and independently evaluate individual subsystems, such as imaging, couch movement, and beam delivery. Another area of ongoing development is the utilization of the onboard MV imaging system to create a similar QA procedure that could eliminate the need for external 2D detector arrays entirely, thereby enabling a fully automated process with minimal human intervention. Additionally, the in‐house iQA software and associated tools developed for this workflow will be made available to other institutions upon reasonable request for noncommercial, academic use to support broader adoption and standardization of automated daily QA practices.

## 5 CONCLUSIONS

This study introduces and validates an automated, comprehensive daily QA solution tailored for the RefleXion X1 PET/CT‐guided linac system. By integrating a custom‐designed IMRT QA plan with a 2D diode detector and in‐house software (iQA), the workflow enables fast and consistent evaluation of key performance metrics including output stability, image guidance accuracy, and couch positioning reliability. The system has been clinically implemented and demonstrated strong performance over a year‐long evaluation, successfully detecting gradual output drifts and providing submillimeter accuracy in offset detection. The end‐to‐end design simplifies QA procedures, reduces human error, and offers scalability for broader clinical adoption. Future developments will focus on incorporating PET QA and rotational couch validations to further enhance the system's comprehensiveness and adaptability.

## AUTHOR CONTRIBUTIONS

All authors contributed significantly to this work. Yang Kyun Park and Bin Cai conceptualized the study, supervised the project, and led manuscript writing. Sagar Ghimire, Grant Gibbard, Jun Tan, Thomas I. Banks, and Chenyang Shen performed data collection and analysis. Sagar Ghimire, Yang Kyun Park, Grant Gibbard, Jun Tan, and Bin Cai contributed to methodology design and manuscript editing. Rameshwar Prasad, Tingliang Zhuang, Andrew Godley, and Steve B. Jiangprovided clinical oversight and critical revisions. All authors reviewed and approved the final manuscript.

## CONFLICT OF INTEREST STATEMENT

BC received research grant, trial support and honorarium from RefleXion Medical outside this wok. The remaining authors declare that the research was conducted in the absence of any commercial or financial relationships that could be construed as a potential conflict of interest

## ETHIC STATEMENT

This study did not involve the use of data from human subjects or animals.

## Data Availability

The original contributions presented in this study are included in the article. Further inquiries can be directed to the corresponding author(s).
